# Evidence of second-order transition and critical scaling for the dynamical ordering transition in current-driven vortices

**DOI:** 10.1038/s41598-024-51534-5

**Published:** 2024-01-12

**Authors:** S. Maegochi, K. Ienaga, S. Okuma

**Affiliations:** Department of Physics, Tokyo Institute of Technology, 2-12-1 Ohokayama, Meguro-ku, Tokyo, 152-8551 Japan

**Keywords:** Physics, Condensed-matter physics

## Abstract

Dynamical ordering from a disordered plastic flow to an anisotropically ordered smectic flow induced by a dc force has been studied in various many-particle systems, including vortices in type-II superconductors. However, it remains unclear whether the dynamical ordering is a true phase transition because of lack of suitable experimental methods. Here, we study the response of vortex flow to the *transverse* force using a cross-shaped amorphous Mo$_{x}$Ge$_{1-x}$ film. From transverse current-voltage (force-velocity) characteristics under various longitudinal currents, we find a change of the transverse response in low voltage (velocity) regions from a nonlinear to linear behavior at a well-defined longitudinal current that marks the dynamical ordering transition. We also find the scaling collapse of the transverse current-voltage curves to a universal scaling function, providing evidence of the second-order transition for the dynamical ordering transition.

## Introduction

When a magnetic field is applied perpendicular to the plane of type-II superconductors, magnetic flux quanta called vortices are generated^1^. By applying a current above the depinning threshold, the vortices pinned by the quenched disorder start to flow due to the Lorentz-like force exerted from the current and their motion causes energy dissipation. Therefore, understanding the depinning and vortex dynamics is of practical importance. Fundamentally, the dynamics of vortices have been intensively studied because they exhibit rich nonequilibrium phases and phase transitions^2–14^, which are generic to many-particle assemblies driven over random substrates^15^. When the driving force is increased and the interaction between the vortices and pinning centers is reduced, the flow structure of the vortices is considered to show dynamical ordering from a disordered plastic flow to an anisotropically ordered smectic flow^16–20^.

One of the long-standing questions is whether the current-induced dynamical ordering from the plastic flow to the smectic flow actually takes place. However, this question has not been answered experimentally because from conventional transport measurements, it is difficult to detect the moving smectic phase with long-range order in the direction transverse to the driving force^16–21^. Recently, we have overcome this problem by using two-step measurements of transient voltage in response to mutually perpendicular driving currents^22^. We found dynamical ordering from the plastic flow to the anisotropic smectic flow as a function of the current. Convincing evidence of the moving smectic phase was obtained from the first transverse mode locking with signals larger than those of longitudinal mode locking, indicating the higher transverse order than the longitudinal one. However, the central issue of whether the current-induced dynamical ordering is a phase transition or a crossover still remains elusive. If it turns out to be a true phase transition, it is also of interest to examine whether it shows a critical behavior.

In this work, we resolve the issue by studying the critical scaling for the dynamical ordering transition. The scaling approach is generally employed to demonstrate second-order phase transitions and critical phenomena^23–25^. Here, we measure the response of vortex flow to the *transverse* driving force using a cross-shaped amorphous Mo$_{x}$Ge$_{1-x}$ film. From transverse current-voltage (i.e., force-velocity) characteristics under various longitudinal currents superimposed with the transverse current, we find a change of the transverse response in low voltage (velocity) regions from a nonlinear behavior with nonzero transverse depinning current to a linear behavior with zero transverse depinning current at a well-defined longitudinal current that marks the dynamical ordering from the plastic to smectic flow. We also find a scaling collapse of the transverse current-voltage curves to a universal scaling function, providing firm evidence of the second-order transition for the dynamical ordering transition.

**Figure 1:**
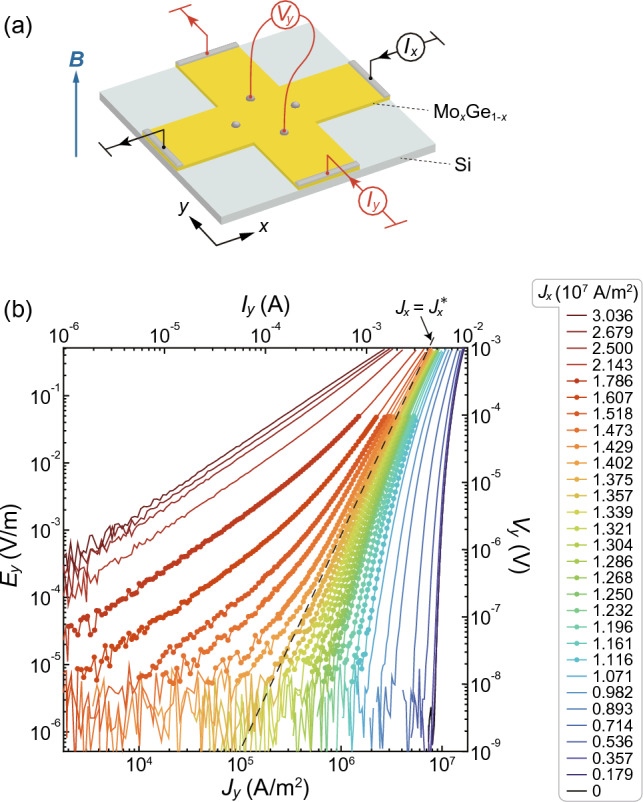
(**a**) Schematics of the experimental setup of the cross-shaped amorphous Mo$_x$Ge$_{1-x}$ film on the Si substrate. The magnetic field *B* is applied perpendicular to the film surface. (**b**) $J_{y}-E_{y}$ characteristics measured under fixed $J_x$ listed on the right-hand side. The right axis indicates the voltage $V_{y}$ in the *y* direction. Solid circles represent the points used for the scaling analysis in Fig. 4. A dashed line labeled $J_{x}=J_{x}^{*}$ shows the power-law behavior.

**Figure 2:**
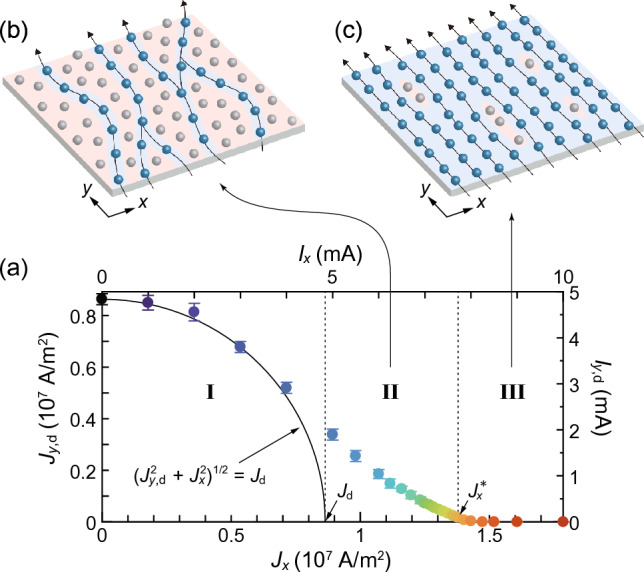
(**a**) $J_{y,\text{d}}$ deduced from the $J_{y}-E_{y}$ curves in Fig. 1b using a $10^{-8}$ V criterion plotted against $J_{x}$. The right axis indicates the depinning current $I_{y,\text{d}}$ in the *y* direction. Vertical dotted lines mark the isotropic depinning current density $J_\text{d}$ and the threshold current density $J_{x}^{*}$, separating the regions I and II and regions II and III, respectively. In region I, all the vortices are pinned initially and then undergo depinning obliquely, when $(J_{y}^2 + J_{x}^2)^{1/2}$ exceeds $J_\text{d}$. A solid quadrant line represents $(J_{y,\text{d}}^2 + J_{x}^2)^{1/2} = J_\text{d}$. In region II, the vortices initially flowing in the longitudinal (*y*) direction undergo the transverse depinning for $J_{y}>J_{y,\text{d}}$. In region III, the transverse depinning does not occur ($J_{y,\text{d}}=0$). (**b**,**c**) Schematics of initial vortex flow generated by $J_{x}$. (b) The plastic flow in region II. (**c**) The smectic flow in region III. The areas shaded by red and blue represent the regions with strong and weak effective pinning, respectively.

## Results and discussion

To study the transverse response of flowing vortices, we used a cross-shaped film of amorphous Mo$_{x}$Ge$_{1-x}$ (see “Methods”), in which we can apply the driving current in *x* and *y* directions simultaneously. The schematic of experimental setup is shown in Fig. 1a. The magnetic field of 1.0 T was applied perpendicular to the plane of the film at 3.6 K to generate the vortices.

We first apply the current (driving force) in the minus *x* direction (*y* direction) with a given current density $J_{x}$ and then examine the response to the transverse driving force applied in the *x* direction by measuring the transverse current-voltage characteristics in the *y* direction as shown with red color in Fig. 1a. Here, $J_{x}$ is the control parameter that changes the flow structure of vortices, while the transverse $J_{y}-E_{y}$ characteristics are used to probe the transverse vortex response, where $J_{y}$ and $E_{y}$ are the current density and electric field in the *y* direction, respectively. Figure 1b displays $J_{y}-E_{y}$ curves in a double logarithmic scale measured under various $J_{x}$. For smaller $J_{x}$, the $J_{y}-E_{y}$ curves have a negative curvature and $E_{y}$ rapidly drops below the voltage resolution of $10^{-8}$ V upon reducing $J_{y}$. At around $J_{x} \approx 1.38\times 10^7$ A/m$^2$ ($\equiv J_{x}^{*}$), the $J_{y}-E_{y}$ curve exhibits a power-law behavior as shown by a dashed straight line. For $J_{x}> J_{x}^{*}$, the $J_{y}-E_{y}$ curves have a positive curvature and cross over to a low-current linear behavior upon reducing $J_{y}$.

From Fig. 1b, we extract the depinning current density $J_{y,\text{d}}$ using a $10^{-8}$ V criterion and plot it against $J_{x}$ in Fig. 2a. It is found that there are three qualitatively different regimes, which are separated by the ordinary depinning current density $J_\text{d}$ and $J_{x}^{*}$, as shown by vertical dotted lines. In the region with $J_{x} < J_\text{d}$ (region I), the vortices are initially pinned before applying $J_{y}$ since $J_{x}$ is smaller than the depinning current density $J_\text{d}$. With an increase in $J_{y}$, the depinning occurs when $J_{y} = \sqrt{J_\text{d}^{2} - J_{x}^{2}}$, as shown by a quadrant line. This indicates the ordinary depinning in the oblique direction by the combined currents of $J_{x}$ and $J_{y}$. In the region where $J_\text{d}< J_{x} < J_{x}^{*}$ (region II), the vortices initially flow in the longitudinal (*y*) direction due to the Lorentz-like force by $J_{x}$. Nevertheless, $J_{y,\text{d}}$ is nonzero, indicating the occurrence of the nontrivial depinning in the transverse (*x*) direction, which we call a transverse depinning^26,27^. For $J_{x} > J_{x}^{*}$ (region III), $J_{y,\text{d}}$ is zero and the vortices can flow freely in the transverse (*x*) direction by infinitesimal $J_{y}$.

The disappearance of the transverse depinning at $J_{x}\ge J_{x}^{*}$ implies the presence of the dynamical transition of the longitudinal vortex flows (in the *y* direction) at $J_{x}^{*}$, where the transverse response changes from a nonlinear behavior with $J_{y,\text{d}}>0$ to a linear behavior with $J_{y,\text{d}}=0$. We have found recently in the same sample that the transition or crossover from the plastic flow to the smectic flow takes place at $J_{x}\approx 1.5\times 10^7$ A/m$^2$^22^. Since this value is close to the value of $J_{x}^{*} \approx 1.38\times 10^7$ A/m$^2$ obtained here, the change of the flow state at $J_{x}^{*}$ is considered to correspond to the dynamical ordering transition from the plastic flow in the region II to the smectic flow in the region III. This view, together with the second-order nature of the transition, is justified by the scaling analysis described below.

As schematically illustrated in Fig. 2b, the plastic flow initially generated by $J_{x} (<J_{x}^{*})$ is a disordered flow dominated by random pinning and shows riverlike features, where a small number of vortices flow around regions of pinned vortices shaded by red color^28,29^. Once such flow patterns are formed in the *y* direction, it is difficult even for the flowing vortices to flow in the transverse (*x*) direction when the small driving force (driving current $J_{y}$) is applied in the *x* direction (*y* direction). This accounts for the nonzero $J_{y,\text{d}}$ in the region II.

In contrast, when $J_{x}$ larger than $J_{x}^{*}$ is applied initially, the effects of pinning are much more suppressed and the smectic flow is generated, as schematically shown in Fig. 2c. In the smectic flow, the vortices form one-dimensional channels along the longitudinal (*y*) direction with long-range transverse order in the *x* direction^16–21^. The areas of pinned vortices (shaded by red color) shrink and instead the pin-free regions (shaded by blue) grow, percolating in the transverse (*x*) direction. As a result, the vortices no longer feel transverse barriers and flow freely in the *x* direction by the infinitesimal driving force ($J_{y}$), which explains the absence of the transverse depinning in the region III.

**Figure 3:**
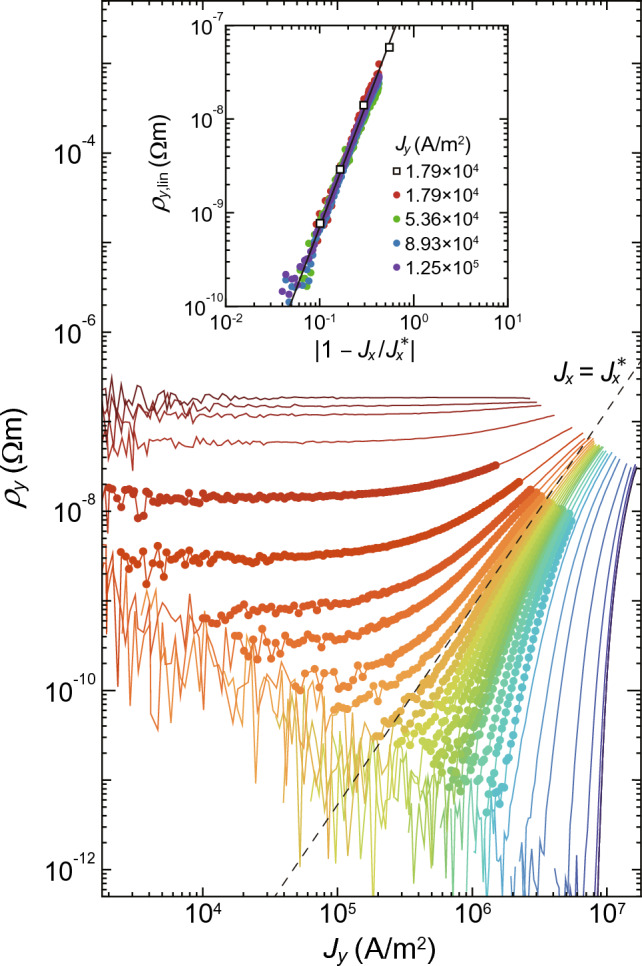
Log-log plots of the $J_{y}-\rho _{y}$ data converted from the $J_{y}-E_{y}$ data in Fig. 1b. A dashed line labeled $J_{x}=J_{x}^{*} (\approx 1.38\times 10^7$ A/m$^2$) indicates the power law of Eq. (2), $\rho _{y} \propto J_{y}^{\beta /\Delta }$ with $\beta /\Delta = 2.2\pm 0.3$. Inset: Log-log plots of the linear resistivity $\rho _{y,\text{lin}}$ as a function of $|1 - J_{x}/J_{x}^{*}|$. Solid circles represent the data from $\rho _{y}(J_{x}, J_{y})$ measured by using small currents, $J_y = 1.79\times 10^4,$ $5.36\times 10^4,$ $8.93\times 10^4,$ and $1.25\times 10^5$ A/m$^2$, with changing $J_{x}$ continuously. Open squares are the data collected from $\rho _{y}(J_{x},J_{y})$ at $J_y = 1.79\times 10^4$ A/m$^2$ in the main panel. A solid straight line indicates the fit to Eq. (3), $\rho _{y,\text{lin}} \propto |1 - J_{x}/J_{x}^{*}|^{\beta }$ with $\beta = 2.65 \pm 0.3$.

**Figure 4:**
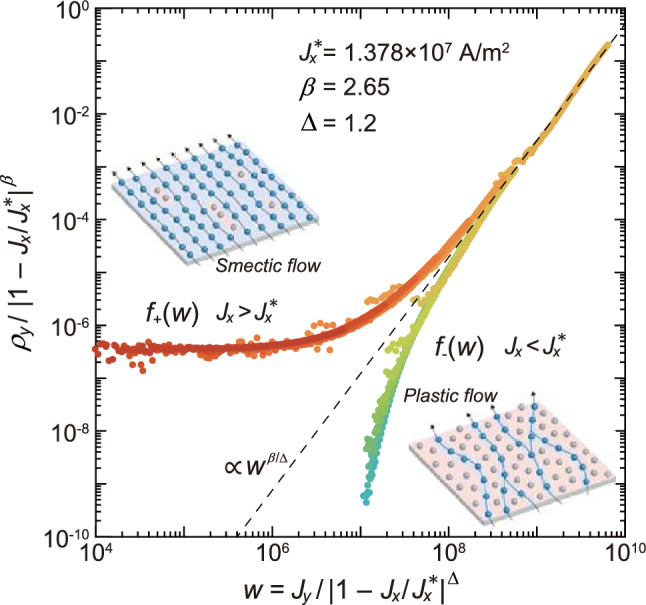
The scaling plot of the transverse $J_{y}-\rho _{y}$ data shown with solid circles in Fig. 3 using Eq. (1): $\rho _{y}(J_{x}, J_{y}) / |1 - J_{x}/J_{x}^{*}|^{\beta }$ versus $w(= J_{y}/|1 - J_{x}/J_{x}^{*}|^\Delta )$. A good collapse of the data to the universal branches is obtained with $J_{x}^{*} = 1.378\times 10^7$ A/m$^2$, $\beta = 2.65$, and $\Delta = 1.2$. A dashed straight line represents the asymptotic behavior for $w\rightarrow \infty$, $\rho _{y} / |1 - J_{x}/J_{x}^{*}|^{\beta }\propto w^{\beta /\Delta }$. Inset: Schematics of the plastic flow ($J_{x}<J_{x}^{*}$) and the smectic flow ($J_{x}>J_{x}^{*}$).

The current-voltage ($J_{y}-E_{y}$) characteristics in Fig. 1b with a sign change of curvature at $J_{x}^{*}$ is reminiscent of the isotherms near the critical point for gases^30^, Ising ferromagnets^25^, and vortex glasses^31–40^, and the flow curves for yielding and jamming transitions^41–43^, where the critical scaling associated with the second-order phase transition has been well established. We perform the scaling analysis for the $J_{y}-\rho _{y}$ characteristics shown in Fig. 3, which are converted from the $J_{y}-E_{y}$ data in Fig. 1b, to test whether the dynamical transition at $J_{x}^{*}$ is the second-order phase transition, where $\rho _{y}\equiv E_{y}/J_{y}$. If the threshold current $J_{x}^{*}$ is a critical point for the second-order transition, the resistivity $\rho _{y}(J_{x}, J_{y})$ as functions of $J_{x}$ and $J_{y}$ should obey the following scaling equation^25,44^:

1 $$\begin{aligned} \rho _{y}(J_{x}, J_{y}) = |1 - J_{x}/J_{x}^{*}|^{\beta } f_{\pm }\left( \frac{J_{y}}{|1 - J_{x}/J_{x}^{*}|^{\Delta }}\right) , \end{aligned}$$

where $\beta$ and $\Delta$ are scaling exponents, $w \equiv J_{y}/|1 - J_{x}/J_{x}^{*}|^\Delta$ is the scaling variable, and $f_{+}(w)$ and $f_{-}(w)$ are the two branches of the scaling function for $J_{x}>J_{x}^{*}$ and $J_{x}<J_{x}^{*}$, respectively. For $w\rightarrow \infty$, the scaling function takes an asymptotic form $f_{\pm }(w) \approx w^{\beta /\Delta }$. This gives a power law $J_{y}-\rho _{y}$ relation at $J_{x} = J_{x}^{*}$, which satisfies scale invariance,

2 $$\begin{aligned} \rho _{y}(J_{x} = J_{x}^{*}) \propto J_{y}^{\beta /\Delta }. \end{aligned}$$

For $w\rightarrow 0$, the two branches are expected to be $f_{-}(w) =0$ and $f_{+}(w) =\mathrm {const.}$, which gives the power-law scaling of a linear resistivity $\rho _{y,\text{lin}}$ for $J_{x}>J_{x}^{*}$,

3 $$\begin{aligned} \rho _{y,\text{lin}} \propto |1 - J_{x}/J_{x}^{*}|^{\beta }. \end{aligned}$$

A dashed line in Fig. 3 represents the power law of Eq. (2) with $\beta /\Delta = 2.2\pm 0.3$. The inset of Fig. 3 displays the log-log plot of $\rho _{y,\text{lin}}$ as a function of the dimensionless distance from the critical point, $|1- J_{x}/J_{x}^{*}|$, where open squares are $\rho _{y,\text{lin}}$ for $J_y = 1.79\times 10^4$ A/m$^2$ extracted from $\rho _{y}(J_{x},J_{y})$ in the main panel. Solid circles are the resistivity $\rho _{y}(J_{x},J_{y})$ for small currents, $J_y = 1.79\times 10^4,$ $5.36\times 10^4,$ $8.93\times 10^4,$ and $1.25\times 10^5$ A/m$^2$, measured with changing $J_{x}$ continuously. The collapse of all data indicates the linear behavior and $\rho _{y,\text{lin}}$ is found to be scaled in the form of Eq. (3) with $\beta = 2.65\pm 0.3$, as shown with a solid straight line. From the values of $\beta /\Delta$ and $\beta$ obtained here, the exponent $\Delta$ is determined to be $\Delta = 1.2\pm 0.2$.

In Fig. 4, we replot the data shown with solid circles in Fig. 3 with respect to the scaled variables of Eq. (1), $\rho _{y}(J_{x}, J_{y}) / |1 - J_{x}/J_{x}^{*}|^{\beta }$ versus $w(= J_{y}/|1 - J_{x}/J_{x}^{*}|^\Delta )$, where $J_{x}^{*} = 1.378\times 10^7$ A/m$^2$, $\beta = 2.65$, and $\Delta = 1.2$ are used. A good scaling collapse to universal branches is found, in agreement with Eq. (1). A dashed straight line represents the asymptotic behavior for $w\rightarrow \infty$, $\rho _{y} / |1 - J_{x}/J_{x}^{*}|^{\beta }\propto w^{\beta /\Delta }$. The results provide convincing evidence that the dynamical ordering transition from the plastic flow to the smectic flow, which occurs at $J_{x}=J_{x}^{*}$, is indeed the second-order phase transition. Recent simulation studying the Kibble-Zurek mechanism for dynamical ordering also predicted the continuous phase transition^45^, which was indirectly supported by our experiment in the vortex system^46^, consistent with the present results. In these studies, the dynamical ordering transition is considered to be an absorbing phase transition in $1+1$ dimensional directed percolation universality class^45–47^.

In the scaling analysis, the probe current $J_y$ up to about 50% of the drive current $J_x$ is used. Unless $J_y$ is sufficiently smaller than $J_x$, the probe $J_y$ may affect the flow state formed by the drive $J_x$. We have confirmed that the possible interference effect of the probe current does not seriously affect our discussion (See Supplementary Material for discussion of the possible interference effect of the probe current on the drive current).

We have shown that with an increase in $J_{x}$, the transverse response of vortex flow changes from the nonlinear behavior associated with the transverse depinning ($J_{x}<J_{x}^{*}$) to the linear one without the transverse depinning ($J_{x}>J_{x}^{*}$). The result together with the scaling collapse indicates that the dynamical ordering transition from the plastic flow to the smectic flow is of second order. The disappearance of the transverse depinning for $J_{x}>J_{x}^{*}$ results from the reduced effective pinning due to increased $J_{x}$. As mentioned above, the present finding is analogous to the vortex-glass transition^31–40^, where the vortex phase below the transition temperature is the vortex-glass phase dominated by pinning, while the high-temperature phase is the vortex-liquid phase, where the pinning is ineffective due to thermal fluctuations. The role of the current $J_{x}$ in the dynamical ordering transition corresponds to that of the temperature in the vortex-glass transition, both of which play a similar role in weakening the pinning effects.

The scaling exponents $\beta = 2.65\pm 0.3$ and $\Delta = 1.2\pm 0.2$ obtained in this work are slightly smaller than those of the vortex-glass transition, $\beta = 4$–8 and $\Delta = 2$–4^31,33^. This discrepancy is not surprising because the two transitions are rather different: The dynamical ordering is the nonequilibrium phase transition and the effect of the current is anisotropic while the vortex-glass transition is the equilibrium phase transition and the effect of the temperature is isotropic.

## Methods

### Sample preparation

The 280-nm-thick cross-shaped amorphous Mo$_x$Ge$_{1-x}$ ($x\approx 0.78$) film with weak random pinning was deposited using rf sputtering onto a Si substrate held at room temperature^22^. Current ($I_{x}, I_{y}$) and voltage ($V_{y}$) electrodes are arranged as schematically shown in Fig. 1a. $V_{x}$ is measured using voltage electrodes arranged in the *x* direction. The size of the central intersection of the sample is $2\times 2$ mm$^2$ and the distance between voltage electrodes is 1.95 mm. The critical temperature $T_\text{c} = 6.2$ K is independent of the directions, indicating the uniformity of the film. The sample was directly immersed in liquid $^4$He to reduce possible heating.

### Transport measurements

We conducted standard four-probe measurements at 3.6 K and 1.0 T, corresponding to the Bragg-glass phase at equilibrium^48^. We confirmed that the depinning current densities $J_{\text{d}}=0.86\times 10^{7}$ A/m$^2$ in the *x* and *y* directions are identical to each other. For the $J_{y}-E_{y}$ measurements, we measured $E_{y}(J_{x},J_{y})$ and $E_{y}(J_{x},J_{y}=0)$ for each $J_{y}$. As a result, we safely subtracted the background signal, including the small component of $E_{x}$ coming from the possible misalignment of voltage electrodes, and obtained reliable values of the transverse voltage $E_{y}$. Compared with the conventional strip-shaped film, some current may leak in a wider central zone in our cross-shaped film. We believe that this may lead to a slight overestimation of the absolute value of currents but does not influence the discussion, in particular, the scaling analysis. Further measurements, such as using samples with the voltage contacts placed closer to the cross center, would prove it clearly.

### Supplementary Information

Supplementary Information.

## Data Availability

The data that support the findings of this study are available from the corresponding author upon reasonable request.
