# Supplementary material for: Evidence of second-order transition and critical scaling for the dynamical ordering transition in current-driven vortices
Source: Sci Rep. 2024 Jan 12;14:1232. doi: 10.1038/s41598-024-51534-5 (PMC10786941; doi:10.1038/s41598-024-51534-5)
Supplement: Supplementary file 1 — Supplementary Information. [file 41598_2024_51534_MOESM1_ESM.pdf]

Evidence of second-order transition and critical scaling for the dynamical ordering transition in current-driven vortices

Supplementary Material

S. Maegochi*, K. Ienaga, and S. Okuma†

Department of Physics, Tokyo Institute of Technology,
2-12-1 Ohokayama, Meguro-ku, Tokyo 152-8551, Japan

(Dated: December 28, 2023)

*maegochi.shun.1014@gmail.com
†sokuma@o.cc.titech.ac.jp

SCALING ANALYSIS

In the scaling plots of the $J_y - \rho_y$ data shown in Fig. 4 in the main text, the probe current J_y up to about 50% of the drive current J_x was used. Unless J_y is sufficiently smaller than J_x, the probe current J_y may affect the flow state formed by the drive current J_x and the interference effect of the probe current cannot be neglected. To test the validity of the scaling behavior under the very small interference effects, we perform the scaling analysis using the data with J_y much smaller than J_x. We show in Figs. S1(a)-(d) the log-log plots of the $J_y - \rho_y$ data measured under fixed J_x listed on the right-hand side of (a). Figures S1(e)-(h) display the scaling plots of the data shown with solid circles in (a)-(d), respectively, which correspond to the data with J_y smaller than (e) 10%, (f) 7%, (g) 5%, and (h) 3% of J_x listed on the right-hand side of each panel.

(1) For $J_x > J_x^*$, a good scaling collapse to a universal function (upper branch) is observed not only for (e), (f), and (g), but also for (h), where $J_y < 0.03J_x$. This is because in the case of $J_x > J_x^*$, $E_y(J_y)$ is well above the experimental resolution even for $J_y < 0.03J_x$. Furthermore, we have confirmed that the power-law scaling of a linear resistivity $\rho_{y,\mathrm{lin}}$ is also observed even for $J_y < 0.03J_x$ as shown in the inset of Fig. 3.

(2) For $J_x < J_x^*$, in contrast, a scaling collapse of the data to a universal function (lower branch) is not visible for (h), where $J_y < 0.03J_x$, since $E_y(J_y)$ falls below the experimental resolution. Nevertheless, for (g), where $J_y < 0.05J_x$, an indication of the scaling collapse is clearly observed, and for (f) and (e), where $J_y < 0.07J_x$ and $J_y < 0.1J_x$, respectively, the scaling behavior which is almost identical to that in Fig. 4 is clearly observed.

(3) The scaling parameters, $J_x^* = 1.378 \times 10^7$ A/m^2, $\beta = 2.65$, and $\Delta = 1.2$, are obtained even from the data for $J_y < 0.03J_x$ as well as from the data for $J_y < 0.1J_x$, which are the same as the values deduced from the data in Figs. 3 and 4.

Note that (i) the degradation of the scaling collapse, that is, the missing and deviation of data points from the scaling curve, observed for $J_x < J_x^*$ and $J_y < 0.03J_x$ originates solely from the sensitivity limit of the E_y measurements; (ii) to our knowledge, there is no strict a priori criterion of J_y/J_x (a ratio of the probe current to the drive current) for the interference effect to be safely neglected. We thus consider that the scaling collapse of data onto the two branches observed for $J_y < 0.05J_x$ and shown in Fig. S1(g) indicates that the interference effect does not seriously affect our conclusion.

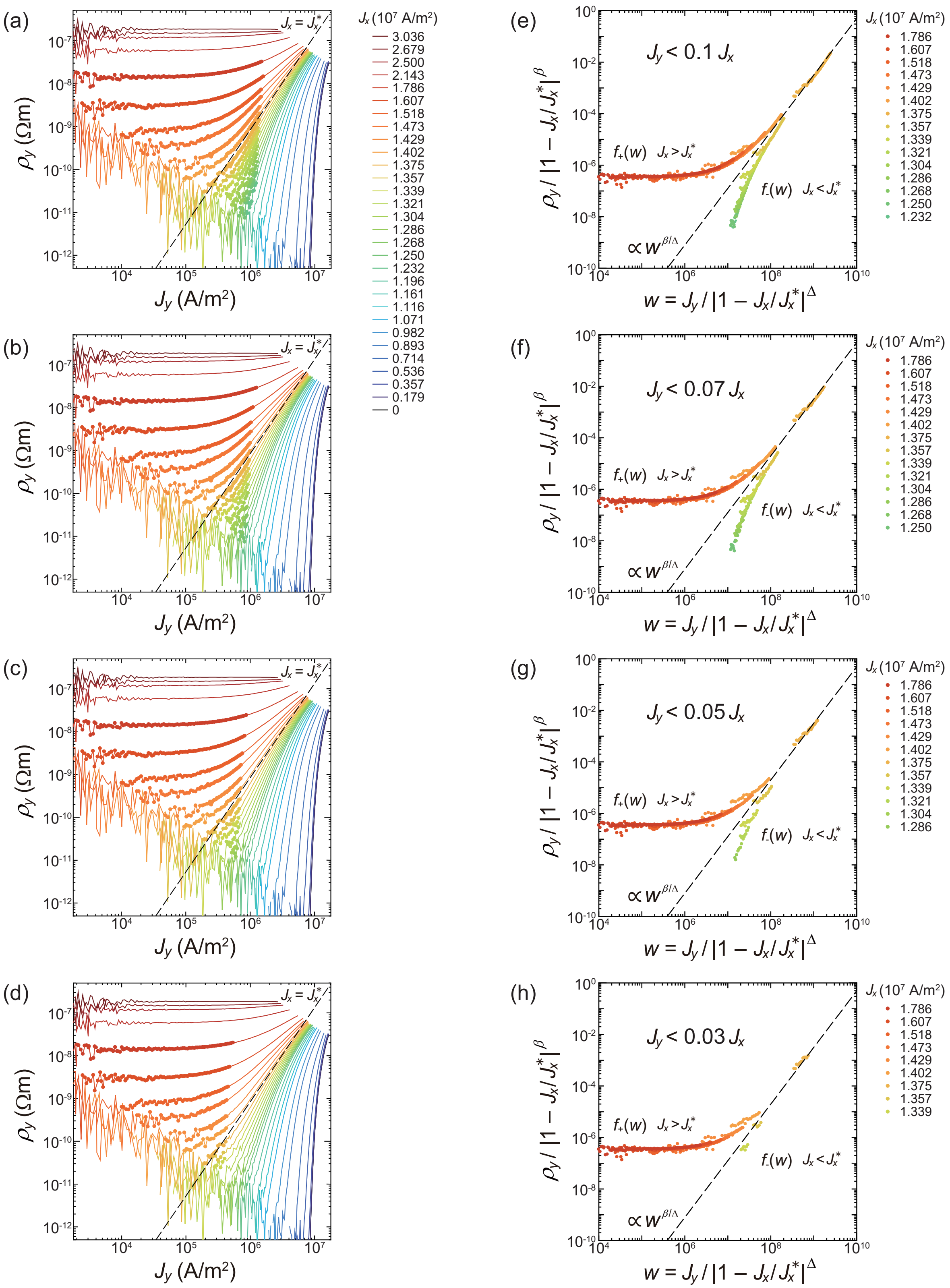

FIG. S1. (a)-(d) Log-log plots of the $J_y - \rho_y$ data measured under fixed J_x listed on the right-hand side of (a). (e)-(h) The scaling plots of the data denoted with solid circles in (a)-(d) are shown in (e)-(h), respectively. In the scaling analysis, we use the data with J_y smaller than (e) 10%, (f) 7%, (g) 5%, and (h) 3% of each J_x listed on the right-hand side. The values of $J_x^* = 1.378 \times 10^7$ A/m^2, $\beta = 2.65$, and $\Delta = 1.2$ are the same as the values obtained in Figs. 3 and 4 in the main text.
